# High Multiplicity Infection by HIV-1 in Men Who Have Sex with Men

**DOI:** 10.1371/journal.ppat.1000890

**Published:** 2010-05-13

**Authors:** Hui Li, Katharine J. Bar, Shuyi Wang, Julie M. Decker, Yalu Chen, Chuanxi Sun, Jesus F. Salazar-Gonzalez, Maria G. Salazar, Gerald H. Learn, Charity J. Morgan, Joseph E. Schumacher, Peter Hraber, Elena E. Giorgi, Tanmoy Bhattacharya, Bette T. Korber, Alan S. Perelson, Joseph J. Eron, Myron S. Cohen, Charles B. Hicks, Barton F. Haynes, Martin Markowitz, Brandon F. Keele, Beatrice H. Hahn, George M. Shaw

**Affiliations:** 1 Department of Medicine, University of Alabama at Birmingham, Birmingham, Alabama, United States of America; 2 Department of Biostatistics, University of Alabama at Birmingham, Birmingham, Alabama, United States of America; 3 Theoretical Biology and Biophysics (T6), Los Alamos National Laboratory, Los Alamos, New Mexico, United States of America; 4 Department of Mathematics and Statistics, University of Massachusetts, Amherst, Massachusetts, United States of America; 5 Nuclear and Particle Physics, Astrophysics and Cosmology (T-2), Los Alamos National Laboratory, Los Alamos, New Mexico, United States of America; 6 Santa Fe Institute, Santa Fe, New Mexico, United States of America; 7 Department of Medicine, University of North Carolina, Chapel Hill, North Carolina, United States of America; 8 Department of Medicine, Duke University, Durham, North Carolina, United States of America; 9 Aaron Diamond AIDS Research Center, New York, New York, United States of America; 10 Rockefeller University, New York, New York, United States of America; 11 SAIC-Frederick, National Cancer Institute, Frederick, Maryland, United States of America; 12 Department of Microbiology, University of Alabama at Birmingham, Birmingham, Alabama, United States of America; NIH/NIAID, United States of America

## Abstract

Elucidating virus-host interactions responsible for HIV-1 transmission is important for advancing HIV-1 prevention strategies. To this end, single genome amplification (SGA) and sequencing of HIV-1 within the context of a model of random virus evolution has made possible for the first time an unambiguous identification of transmitted/founder viruses and a precise estimation of their numbers. Here, we applied this approach to HIV-1 *env* analyses in a cohort of acutely infected men who have sex with men (MSM) and found that a high proportion (10 of 28; 36%) had been productively infected by more than one virus. In subjects with multivariant transmission, the minimum number of transmitted viruses ranged from 2 to 10 with viral recombination leading to rapid and extensive genetic shuffling among virus lineages. A combined analysis of these results, together with recently published findings based on identical SGA methods in largely heterosexual (HSX) cohorts, revealed a significantly higher frequency of multivariant transmission in MSM than in HSX [19 of 50 subjects (38%) versus 34 of 175 subjects (19%); Fisher's exact p = 0.008]. To further evaluate the SGA strategy for identifying transmitted/founder viruses, we analyzed 239 overlapping 5′ and 3′ half genome or *env*-only sequences from plasma viral RNA (vRNA) and blood mononuclear cell DNA in an MSM subject who had a particularly well-documented virus exposure history 3–6 days before symptom onset and 14–17 days before peak plasma viremia (47,600,000 vRNA molecules/ml). All 239 sequences coalesced to a single transmitted/founder virus genome in a time frame consistent with the clinical history, and a molecular clone of this genome encoded replication competent virus in accord with model predictions. Higher multiplicity of HIV-1 infection in MSM compared with HSX is consistent with the demonstrably higher epidemiological risk of virus acquisition in MSM and could indicate a greater challenge for HIV-1 vaccines than previously recognized.

## Author Summary

Understanding the biology of sexual transmission of HIV-1 could contribute importantly to the development of effective prevention measures. However, different routes of virus transmission (vaginal, rectal, penile or oral) and inaccessibility of tissues at or near the time of virus transmission make this goal elusive. Here, we apply single genome amplification and sequencing of plasma HIV-1 and a model of random virus evolution to a cohort of acutely infected men who have sex with men (MSM) and find that MSM are twice as likely as heterosexuals to become infected by multiple viruses as opposed to a single virus. Some MSM subjects were infected by as many as 7 to 10 or more genetically distinct viruses as a consequence of a single exposure event. We go on to molecularly clone the first full-length transmitted/founder subtype B HIV-1 virus and show that it is highly replicative in human CD4+ T-cells but not macrophages. Our study provides the first comparative, quantitative analysis of the multiplicity of HIV-1 infection in the two primary risk groups—MSM and heterosexuals—driving the global pandemic, and we discuss the implications of the findings to HIV-1 vaccine development and prevention research.

## Introduction

An effective sterilizing HIV-1 vaccine ideally should target virus in the earliest stages of transmission, prior to dissemination and establishment of persistent infection [1], [2], [3], [4]. To be broadly protective, such a vaccine must defend against a genetically diverse set of viruses transmitted by different sexual practices and risk behaviors. Results from the recently reported ‘Thai Trial’ RV144 of an experimental HIV-1 vaccine showed a decrease in virus acquisition of 31.2% (p = 0.04) based on a modified intention-to-treat analysis and a trend for greater vaccine effectiveness in those subjects identified as practicing lower risk behaviors [5]. These findings suggest that an HIV-1 vaccine might be more efficacious in preventing infection by some exposure routes than others [5], [6], [7].

Recently, we and others employed SGA, direct sequencing, and a model of random virus evolution to identify those viruses responsible for transmission and productive clinical infection in several largely heterosexual cohorts with acute HIV-1 subtype A, B or C infection [8], [9], [10], [11], [12] and in Indian rhesus macaques inoculated intra-rectally with SIVmac251 or SIVsmmE660 [13]. This experimental approach allows for the distinction of transmitted/founder viruses that differ by as little as a single nucleotide [10], [13]. SGA-direct sequencing also makes possible the identification of transmitted viral sequences in linked transmissions, thereby enabling the unambiguous tracking of viruses from donor to recipient across mucosal surfaces [9], [13], and the molecular cloning and analysis of those viruses actually responsible for productive clinical infection [12].

Previous studies based on different experimental approaches have been informative with respect to determining the overall extent of viral diversity present in acute and early infection as a surrogate for identifying and quantifying transmitted viruses [14], [15], [16], [17], [18], [19], [20], [21], [22], [23], [24]. Such studies generally described new infections as being either “homogeneous,” presumably reflecting infection by one or few viruses, or “heterogeneous,” suggesting infection by more viruses. Based on these studies, a substantial bottleneck in virus transmission was recognized to exist, since the genetic complexity of viral quasispecies in the blood of chronically infected individuals was generally much greater than that in acutely infected subjects. Evidence for a bottleneck in virus transmission, although not necessarily at the mucosal interface, was further suggested by the longstanding observation that most new infections are caused by R5 tropic viruses and not by X4 tropic viruses, which are common in chronic infection [25], [26], [27]. These studies and others in the related Indian rhesus macaque-SIV infection model [13], [28], [29], [30], [31] thus focused attention on the mucosa and submucosa as a potentially important barrier to HIV-1 transmission and a site where critical early virus-host cell interactions leading to transmission and productive clinical infection likely take place [1], [2], [3], [4], [25], [26], [32]. However, it was not until the application of SGA, direct amplicon sequencing, and a model of random virus evolution to the analysis of viral genomes in the acute infection period that actual transmitted/founder viruses could be identified and their numbers precisely estimated [10], [13]. In the present study, we have applied this strategy to a systematic analysis and comparison of multiplicity of HIV-1 infection in men who have sex with men (MSM) versus heterosexuals (HSX).

## Results

### Study Subjects

SGA-direct sequencing was used to identify and enumerate transmitted/founder *env* sequences in 28 acutely infected MSM subjects who reported homosexual exposure as their primary HIV-1 risk behavior and who denied injection drug use (IDU) (**Table 1**). At the time of study, 14 subjects were HIV-1 ELISA negative/western immunoblot (WB) negative (Fiebig stage II), 2 were ELISA+/WB− (Fiebig stage III), 6 were ELISA+/WB indeterminate (Fiebig stage IV) and 6 were ELISA+/WB+/p31− (Fiebig stage V) [10], [33]. Subjects were identified based on clinical symptoms of an acute retroviral syndrome, routine HIV testing in a health care setting, or contact tracing of an HIV-1 infected index case. Clinical histories of sexually transmitted diseases were not available. Envelope sequences from chronically infected sexual partners of two acutely infected subjects were also evaluated.

**Table 1:** Demographics, risk group and baseline laboratory data.

Subject	Subtype	Geographic location	Sexual partners	Sample date	Viral load RNA/ml	CD4 count cells/µl	ELISA^a^	Western blot	Fiebig stage
04013171	B	New York	Multiple	2/6/02	3,700,000	213	R	indet	4
04013211	B	New York	Multiple	8/23/02	19,900,000	846	R	neg	3
04013226	B	New York	Single	11/20/02	26,700,000	175	N	neg	2
04013240	B	New York	Multiple	1/21/03	2,240,000	297	N	neg	2
04013242	B	New York	Multiple	1/23/03	5,790,000	251	R	indet	4
04013291	B	New York	Multiple	6/4/03	1,490,000	179	R	pos(p31-)	5
04013296	B	New York	Multiple	8/5/03	8,050,000	395	N	neg	2
04013321	B	New York	Single	10/10/03	6,250,000	407	N	neg	2
04013327	B	New York	Single	1/27/04	8,720,000	248	R	indet	4
04013383	B	New York	Multiple	4/5/05	584,000	531	N	neg	2
04013396	B	New York	Multiple	8/16/05	1,600,000	581	R	indet	4
04013419	B	New York	Multiple	3/14/06	21,200,000	226	N	neg	2
04013440	B	New York	Multiple	10/17/06	>100,000	205	N	neg	2
04013446	B	New York	Single	11/28/06	>100,000	438	R	neg	3
04013448	B	New York	Single	1/19/07	28,600,000	536	N	neg	2
AD17	B	New York	Multiple	6/14/99	47,600,000	nos	N	neg	2
AD75	B	New York	Multiple	11/6/02	21,400,000	nos	N	neg	2
AD77	B	New York	Multiple	11/15/02	130,000	nos	R	pos(p31-)	5
AD83	B	New York	Multiple	1/22/03	448,000	nos	R	pos(p31-)	5
HOBR0961	B	Alabama	Single	10/31/91	599,238	794	N	neg	2
INME0632	B	Alabama	Single	8/9/90	2,217,670	739	N	neg	2
701010055	B	North Carolina	nos^b^	10/5/06	31,513,812	432	N	neg	2
701010068	B	North Carolina	Multiple	10/24/06	3,714,386	109	R	indet	4
700010106	B	North Carolina	nos	10/19/06	84,545,454	277	N	neg	2
701010027	B	North Carolina	nos	8/29/06	194,744	542	R	pos(p31-)	5
701010108	B	North Carolina	nos	6/28/07	14,711	592	R	pos(p31-)	5
700010246	B	North Carolina	nos	6/7/07	4,395,721	1012	R	indet	4
700010238	B	North Carolina	nos	5/8/07	596,908	587	R	pos(p31-)	5

a R - reactive; N - nonreactive.

b nos - not otherwise specified.

### HIV-1 Env Diversity Analysis

**Figure 1:**
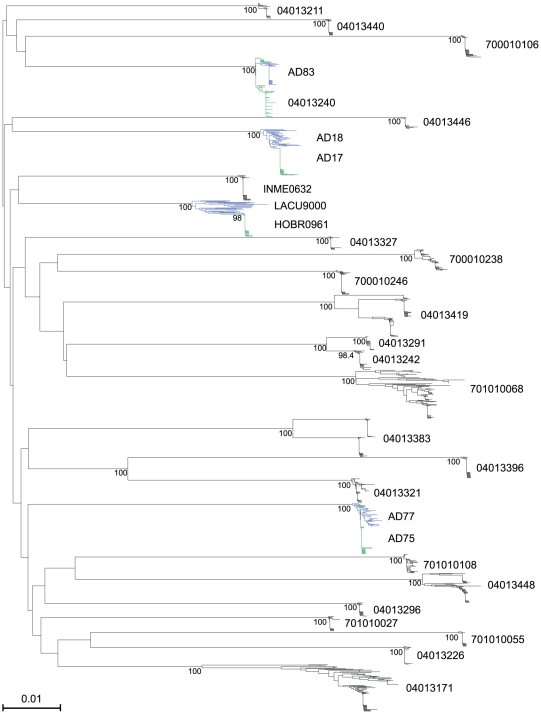
Neighbor-joining (NJ) tree of full-length HIV-1 gp160 env sequences from 28 acutely infected subjects and 2 chronically infected sexual partners. Two chronic-to-acute (LACU9000 to HOBR0961 and AD18 to AD17) and two acute-to-acute (AD77 to AD75 and AD83 to 04013240) transmissions were documented, with donor sequences shown in blue and recipient sequences shown in green. Individual sequences with APOBEC G-to-A hypermutation were excluded from the analysis. Bootstrap values (≥70%) are shown for intra-subject clusters, partner pairs, and additional sequences with evidence of epidemiologic linkage. The horizontal scale bar represents 1.0% genetic distance.

**Figure 2:**
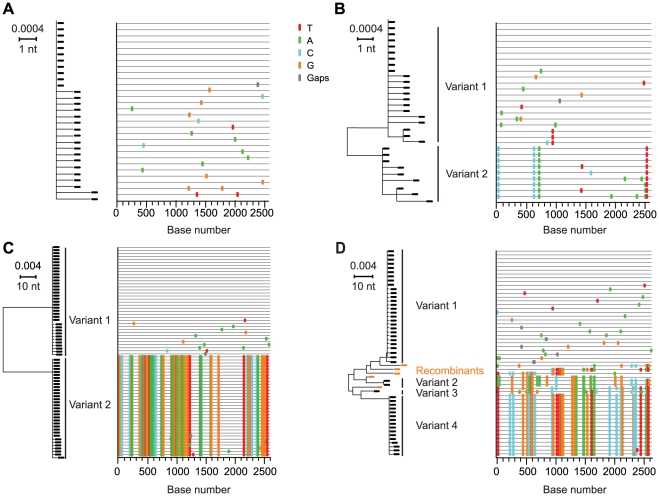
NJ trees and *Highlighter* plots of HIV-1 *env* diversity. Full-length gp160 *env* sequences from four subjects are depicted by NJ tree phylogenies and by *Highlighter*, a sequence visualization tool that allows tracing of common ancestry between sequences based on individual nucleotide polymorphisms [10]. Sequences from subject 04013440 (**A**) showed productive infection by a single virus, from subject 04013211 (**B**) infection by two closely related viruses, from subject 04013383 (**C**) infection by two distantly related viruses, and from subject 04013448 (**D**) infection by four viruses with inter-lineage recombinants denoted by orange symbols in the NJ tree. The horizontal scale bar represents genetic distance.

A total of 1307 full-length *env* genes encoding gp160 were sequenced from plasma vRNA (median of 40 sequences per subject; range 23–89). In a composite neighbor-joining (NJ) phylogenetic tree (**Fig. 1**), viral sequences formed distinct patient-specific monophyletic lineages, each with high statistical support. Sequences from known sexual partners, including two acute-to-acute (AD77 to AD75 and AD83 to 04013240) and two chronic-to-acute (LACU9000 to HOBR0961 and AD18 to AD17) transmission pairs, also clustered significantly together (**Fig. 1**). All sequences were HIV-1 subtype B. Among the 28 acutely infected subjects, maximum within-patient *env* diversities ranged from 0.12% to 6.82% (**Table 2**). Sequences from 22 of these individuals had distinctly lower *env* diversities (<0.75%) compared with *env* diversities from six others (>1.25%). The latter diversity is inconsistent with single virus transmission within the time frame of acute and early infection (Fiebig stage I–V) [8], [9], [10], [34], while *env* diversity <0.75% is consistent either with single variant transmission or with transmission of two or more closely related viruses. Phylogenetic and *Highlighter* analyses of *env* sequences distinguished between these possibilities for each subject (**Fig. 2**; see also www.hiv.lanl.gov/content/sequence/HIV/USER_ALIGNMENTS/Li). **Fig. 2A** shows sequences from a subject (04013440) who was infected by a single virus, **Fig. 2B** a subject (04013211) infected by two viruses differing by only 4 nucleotides out of 2619 (0.15%), **Fig. 2C** a subject (04013383) infected by two viruses differing by 65 of 2547 nucleotides (2.55%), and **Fig. 2D** a subject (04013448) infected by four viruses differing by as many as 47 of 2655 nucleotides (1.79%) with additional sequences showing recombination between the transmitted/founder lineages. Altogether, we determined that 10 of 28 subjects (36%) had been productively infected by more than one virus (**Table 2**).

**Table 2:** Diversity and model analysis of full length *env* sequences from 28 acutely infected subjects.

Subject	Fiebig stage	No. of SGA *envs*	Maximum nt length of *env*	Maximum Hamming distance (HD)^a^	Maximum diversity %	APOBEC-mediated hypermutation	Poisson estimated days since MRCA^b^ (95% CI)	Lambda^c^	Goodness of fit P value^d^	HD fit to poisson	Star phylogeny^e^	Explanation for deviation from model	No. of transmitted/founder viruses	Recombinants^f^
04013171	4	23^g^/86^h^	2625	179	6.82%	No	662 (525, 792)	41.370	0.000	no	no	multiple variant transmission	≥10	20/86
04013211	3	30/30	2619	9	0.34%	No	54 (44, 64)	3.380	0.000	no	no	multiple variant transmission	2	0/30
04013226	2	33/33	2574	5	0.19%	Yes	15 (9, 21)	0.936	0.825	yes	yes		1	
04013240	2	33/66	2568	14	0.55%	No	66 (56, 76)	4.041	0.000	no	no	multiple variant transmission	3	6/66
04013242	4	37/37	2580	5	0.19%	Yes	16 (10, 22)	0.992	0.902	yes	yes		1	
04013291	5	25/25	2559	8	0.31%	No	61 (53, 70)	3.737	0.100	no	no	CTL	1	
04013296	2	25/25	2640	7	0.27%	No	34 (26, 42)	2.113	0.859	yes	yes		1	
04013321	2	49/49	2556	8	0.31%	Yes	39 (33, 45)	2.370	0.212	yes	no	early stochastic mutations	1	
04013327	4	24/24	2535	4	0.16%	No	11 (5, 18)	0.667	0.390	yes	yes		1	
04013383	2	23/70	2547	70	2.75%	No	554 (534, 572)	33.580	0.000	no	no	multiple variant transmission	2	0/70
04013396	4	39/39	2577	5	0.19%	No	16 (11, 21)	0.974	0.897	yes	yes		1	
04013419	2	27/78	2565	80	3.12%	Yes	548 (506, 591)	33.500	0.000	no	no	multiple variant transmission	3	4/78
04013440	2	30/30	2580	4	0.16%	No	23 (18, 28)	1.379	0.116	yes	yes		1	
04013446	3	23/23	2594	7	0.27%	No	24 (12, 35)	1.450	0.000	no	no	early stochastic mutations	1	
04013448	2	15/54	2625	51	1.94%	No	411 (367, 456)	25.716	0.000	no	no	multiple variant transmission	4	5/54
AD17	2	51/51	2544	4	0.16%	Yes	8 (5, 11)	0.471	0.162	yes	yes		1	
AD75	2	54/54	2556	3	0.12%	Yes	9 (6, 13)	0.555	0.774	yes	yes		1	
AD77	5	40/40	2556	11	0.43%	No	84 (74, 95)	5.127	0.151	no	no	multiple variant transmission	3	nd^i^
AD83	5	44/44	2568	33	1.29%	Yes	66 (36, 95)	4.000	0.000	no	no	multiple variant transmission	3	3/44
HOBR0961	2	42/42	2586	4	0.15%	No	17 (13, 22)	1.022	0.503	yes	yes		1	
INME0632	2	46/46	2580	4	0.16%	No	12 (8, 16)	0.737	0.845	yes	yes		1	
701010055	2	28/28	2544	3	0.12%	No	15 (10, 21)	0.923	0.590	yes	yes		1	
701010068	4	17/89	2595	115	4.43%	No	688 (583, 792)	42.499	0.000	no	no	multiple variant transmission	7	30/72^j^
700010106	2	40/40	2595	5	0.19%	Yes	13 (8, 18)	0.800	0.634	yes	yes		1	
701010027	5	27/27	2571	8	0.31%	No	42 (32, 52)	2.552	0.977	yes	yes		1	
701010108	5	35/35	2553	6	0.24%	Yes	39 (34, 45)	2.376	0.099	no	no	CTL	1	
700010246	4	45/45	2607	6	0.23%	Yes	19 (13, 25)	1.180	0.574	yes	yes		1	
700010238	5	38/38	2595	19	0.73%	No	161 (149, 174)	9.970	0.000	no	no	multiple variant transmission	3	8/38

a HD, Hamming Distance - number of base positions at which two sequences differ.

b MRCA - most recent common ancestor (95% confidence interval).

c Lamda - mean of the best fitting Poisson found through maximum likelihood method.

d Goodness of fit P value - χ^2^ goodness-of-fit test statistic for λ, where p<0.05 suggests that the observed distribution of mutations is inconsistent with a Poisson.

e Star phylogeny - random virus evolution.

f Recombinants in gp160 *env*.

g Initial sequence set used for statistical comparisons.

h Total number of sequences analyzed.

i nd - not done.

j Recombinants in gp160 were 30 out of 72 sequences but in the 3′ half genome were 63 out of 72 sequences.

### Model Analysis of HIV-1 Diversification

**Figure 3:**
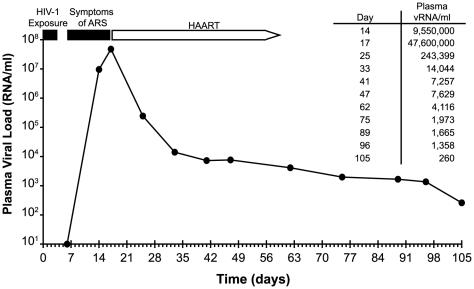
Time course of HIV-1 exposure, symptom onset, viral kinetics, and initiation of antiretroviral therapy in subject AD17. ARS, acute retroviral syndrome. HAART, highly active antiretroviral therapy. For purposes of mathematical modeling, a plasma virus load of 10 RNA molecules per milliliter was estimated at day 6.

**Figure 4:**
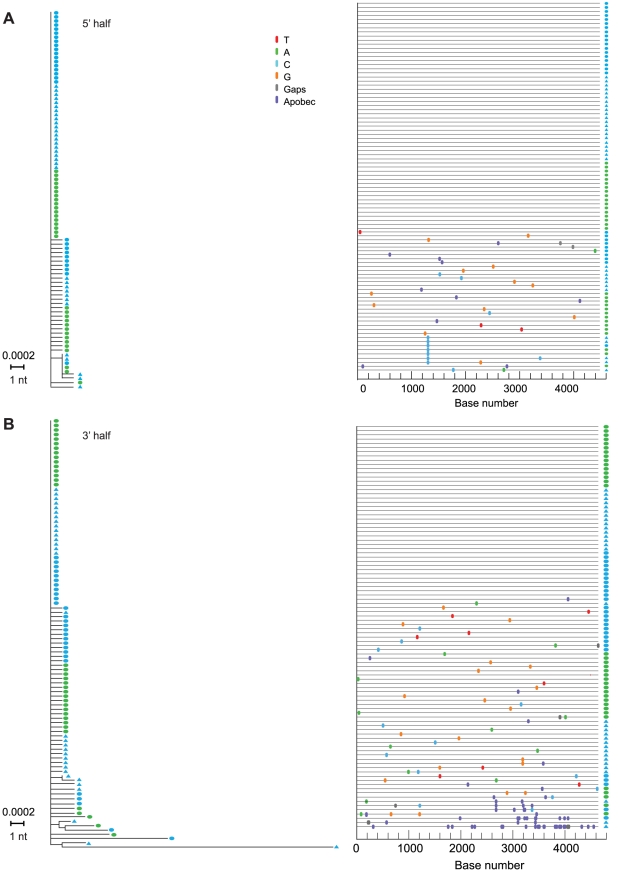
NJ trees and Highlighter plots of HIV-1 diversity in 5′ and 3′ half genomes in subject AD17. Sequences in (**A**) and (**B**) were derived from overlapping 5′ and 3′ SGA amplicons spanning the complete viral genome. Blue symbols represent sequences from day 14 and green symbols day 17 as depicted in **Fig. 3**. Solid ovals represent sequences derived from plasma vRNA and solid triangles represent sequences derived from peripheral blood mononuclear cell DNA.

**Figure 5:**
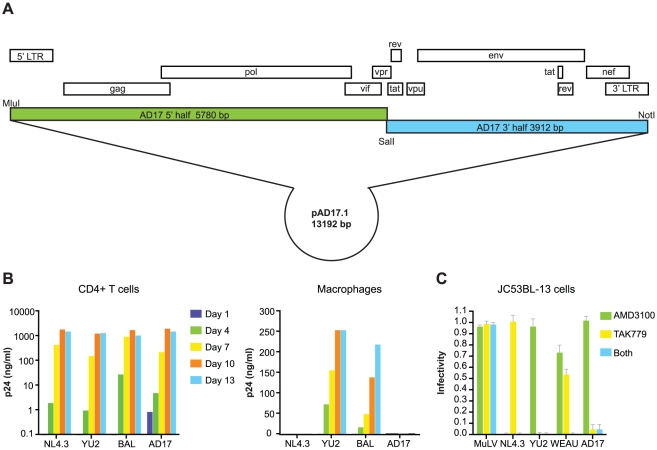
Molecular cloning and biological analysis of the transmitted/founder virus from subject AD17. (**A**) Cloning strategy and genome organization of the transmitted/founder HIV-1 provirus pAD17.1. (**B**) Replication of pAD17.1 virus in activated primary human CD4+ lymphocytes (left panel) and monocyte-derived macrophages (right panel) from the same normal blood donor. Results were replicated three times in cells from different donors, each time showing efficient replication of pAD17.1 virus in CD4+ T cells but not in macrophages. (**C**) pAD17.1 virus infection of JC53BL-13 cells assessed by luciferase expression [12] in the absence or presence of the CXCR4 inhibitor AMD3100 (1.2 uM) or the CCR5 inhibitor TAK779 (10 uM) or both. Results from four experiments are expressed as infectivity (mean ±1 S.D.) relative to control wells lacking coreceptor inhibitor: NL4.3 is X4 tropic, YU2 is R5 tropic, WEAU1.60 is dual R5/X4 tropic, and pAD17.1 is R5 tropic.

We next analyzed the *env* sequences using a mathematical model of random virus evolution that we previously described [10], [13], [34]. Sequences resulting from multivariant transmission, APOBEC hypermutation, early stochastic mutation, selection by cytotoxic T-cells, or recombination violate model predictions (**Table 2**) [10], [34]. Once these confounders were accounted for, lineage-specific *env* sequences from each subject conformed to model predictions and coalesced to most recent common ancestor sequences at or near the time of virus transmission estimated from clinical histories and laboratory staging. These results thus corroborated a large body of evidence supporting the SGA-direct sequencing strategy for identifying transmitted/founder viruses [8], [9], [10], [11], [12], [13], [35]. As an additional test of the model's validity, we asked in subject AD17 whose history of virus exposure was particularly well-documented (**Table 1** **and** **Fig. 3**), if plasma vRNA and PBMC viral DNA (vDNA) sequences spanning the complete (9.2 Kb) viral genome coalesced to the same viral sequence as did *env*-only sequences and if a molecular clone of this viral genome encoded replication competent virus, as would be expected for an authentic transmitted/founder virus. For this analysis, we used SGA-direct sequencing to determine *env*-only sequences (n = 51) and overlapping 5′ (n = 92) and 3′ (n = 96) half genome sequences (**Fig. 4**). All 239 vRNA and vDNA sequences coalesced to a single transmitted/founder genome in a time frame consistent with the clinical history of virus exposure as recently as 11 days earlier. The estimated time to a most recent common ancestor (MRCA) sequence for *env*-only sequences was 8 days (95% CI 5–11) and for all sequences 6–11 days (CI 3–14). MRCA estimates are frequently lower than clinical estimates [10] or experimentally determined intervals between transmission and virus sampling in the rhesus macaque-SIV infection model [13] because of purifying selection or variance in estimated parameters of virus replication [34]. The inferred transmitted/founder viral genome in subject AD17 contained intact LTR-*gag-pol-vif-vpr-tat-rev-vpu-env-nef*-LTR elements, a finding we have replicated for transmitted/founder viruses from 38 other subjects infected by HIV-1 subtypes A, B, C or D ([12] and H.L. and G.M.S., unpublished). A proviral clone (pAD17.1) of the transmitted/founder viral genome from subject AD17 (**Fig. 5A**), when transfected into 293T cells, produced virions that were infectious and highly replicative in human CD4+ T-cells, but interestingly, not in monocyte-derived macrophages from the same normal donors (**Fig. 5B**). pAD17.1 virus was CCR5 tropic in JC53BL-13 cells (**Fig. 5C**) and in GHOST(3) cells [36], where it infected cells bearing CD4 and CCR5 but not CD4 and CXCR4 (G.M.S., unpublished).

### High Multiplicity Infection Followed by Recombination

**Figure 6:**
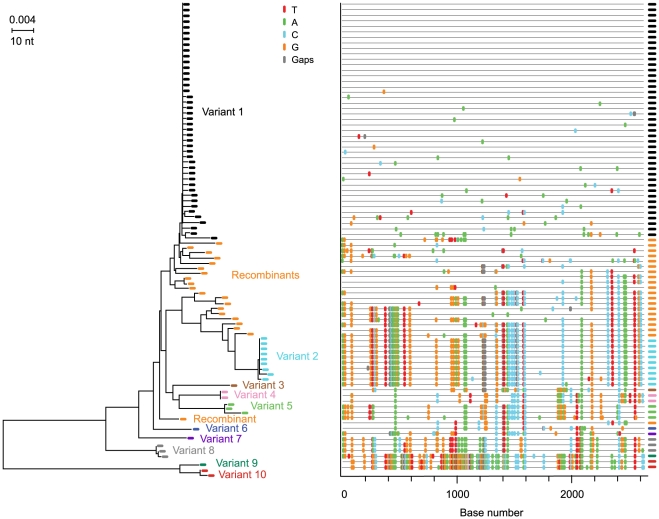
NJ tree and *Highlighter* plot of HIV-1 *env* diversity in subject 04013171. Sequences emanating from ten transmitted/founder viruses are color-coded and identified as variants 1–10. Inter-lineage recombinants are depicted in orange. The horizontal scale bar represents genetic distance.

**Figure 7:**
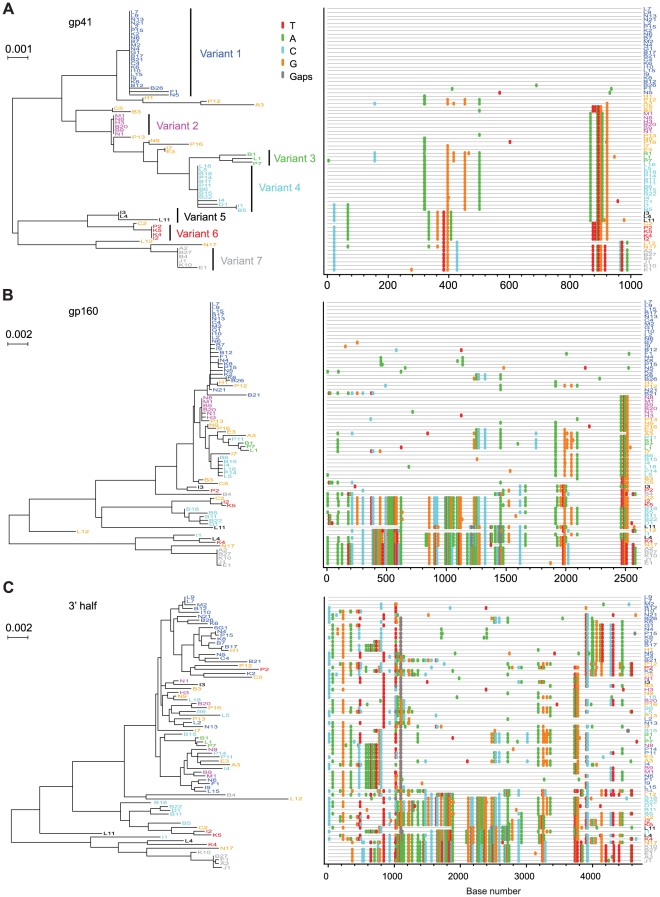
NJ trees and *Highlighter* plots of HIV-1 diversity in *env* gp41, *env* gp160 and 3′ half genomes in subject 7010100068. Seventy-two 3′ half genomes were amplified and sequenced and segments of each are represented in panels **A** (*env* gp41), **B** (*env* gp160) and **C** (3′ half genome). The progeny of transmitted/founder viruses are color-coded and identifiable as discrete ‘rakes’ of identical or nearly identical sequences (variants 1–7) in the *env* gp41 segments shown in panel **A**. The relatedness of sequences emanating from the seven transmitted/founder viruses is progressively obscured in panels **B** and **C** as longer segments are compared due to inter-lineage recombination. The horizontal scale bar represents genetic distance.

Extremes in HIV-1 diversity in acute infection could be informative regarding biological events underlying virus transmission. Subject 04013171 had the greatest *env* diversity (6.82%) (**Table 2**). This subject admitted to unprotected receptive anal intercourse with multiple partners over a single eight hour period four weeks before the onset of flu-like symptoms, consistent with his Fiebig IV staging. **Fig. 6** shows a NJ tree and *Highlighter* plot of 86 plasma derived *env* sequences, which revealed 10 unique transmitted/founder virus lineages. In addition, 20 inter-lineage recombinants were identified based on shared polymorphisms in the *Highlighter* plot with corroboration by Recco analysis [37]. Among these recombinant sequences, the Hudson-Kaplan test [38] indicated a minimum of 44 recombination breakpoints. Interestingly, sequences corresponding to 4 of the 10 virus lineages in subject 04013171 were sampled only once. We could be confident that these represented unique transmitted/founder viruses and not recombinants between two or more predominant virus lineages because of the large number of unique nucleotide changes in each sequence that far exceeded the diversity observed empirically [8], [9], [10], [13] or estimated to occur based on mathematical modeling of the first 35 days of infection (eclipse phase to the end of Fiebig stage IV) [10]. Power calculations further indicated that with a sample size of 86 sequences, there is a >95% probability of detecting minor sequences representing at least 4% of the population (see Methods). These findings suggest that more extensive sampling might result in the detection of an even greater number of transmitted/founder viruses in this individual. Subject 701010068 had the second highest *env* diversity (4.43%) among the study subjects (**Table 2**). He reported a single high risk exposure event involving unprotected receptive anal intercourse with two individuals, one HIV negative and the other HIV positive. He developed flu-like symptoms approximately two weeks later and was studied three weeks after that, again at Fiebig stage IV. Based on the earlier analysis of subject 04013171 (**Fig. 6**), we were concerned that viral recombination [39], [40], [41], [42], which is sequence length and time (from infection) dependent [10], [13], [40], could confound the identification of discrete transmitted/founder virus lineages. This could be especially problematic in subjects infected with many different transmitted/founder viruses as opposed to two, since in the former case it is far more likely that doubly or multiply infected cells will spawn heterozygous virus progeny that lead in the next virus generation to recombinant viral genomes [39], [40], [41]. To test this hypothesis, we amplified and sequenced seventy-two 3′ half genome segments of plasma vRNA from subject 701010068 and then analyzed *env* gp41 (1035 bp), *env* gp160 (2630 bp), and 3′ half genome regions (4734 bp) separately. The gp41 sequences (**Fig. 7A**) revealed discrete low diversity lineages comprised of identical or nearly identical sequences. We interpreted 7 of these sequence clusters as likely to have arisen from distinct transmitted viruses and the remaining sequences to represent inter-lineage recombinants. Clusters of identical or nearly identical sequences were also evident in gp160 sequences (**Fig. 7B**), but with less clarity due to additional inter-lineage recombination events in the longer sequences. For example, sequences corresponding to lineage 4 in the gp41 sequences (depicted in light blue in **Fig. 7A**) were dispersed into five widely separated branches in the gp160 tree due entirely to recombination (**Fig. 3B**). Similarly, sequences comprising lineage 6 in the gp41 sequences (depicted in red in **Fig. 7A**) were dispersed into three widely separated branches in the gp160 tree, again due entirely to recombination (**Fig. 7B**). These findings were supported by Hudson-Kaplan analysis [38], which indicated a minimum of 27 recombination breakpoints among the gp160 *env* sequences. Interspersion of sequences was even more dramatic in the 3′ half genome tree (**Fig. 7C**). Remarkably, of the 72 3′ half genome sequences depicted in **Fig. 7C**, 63 (88%) represented overt recombinants between two or more transmitted/founder lineages demonstrable by visual inspection and by computer-assisted algorithms. Only two (dark blue) sequences labeled L7 and L9 at the very top of the tree (**Fig. 7C**), three (green) sequences labeled B1, L1 and P7 in the middle of the tree, and four (gray) sequences labeled B27, E1, A2 and J1 at the very bottom of the tree showed no evidence of recombination. These findings, together with corroborating data from *env*-only sequences [8], [10], [11], lead to the surprising conclusion that by the time of first antibody detection in acute HIV-1 infection (Fiebig stages III/IV), a majority of circulating viruses may be recombinants. This finding is testament to the large number of doubly (or multiply) infected cells in acute and early infection and further evidence of the rapidity with which virus diversifies [43], [44], making clear that in order to identify non-recombinant transmitted/founder HIV-1 (or SIV) genomes [10], [12], [13], it is necessary to characterize viral sequences as close to the transmission event as possible.

### Comparisons of Multiplicity of HIV-1 Infection in MSM versus Heterosexuals

Four studies, including the present one, have estimated the numbers of viruses responsible for transmission and productive HIV-1 infection after heterosexual or homosexual exposure using identical SGA-direct *env* amplicon sequencing methods [8], [9], [10]. One of these evaluated the frequency of multivariant transmission in a cohort of cohabitating HIV-1 discordant (antiretroviral naïve) heterosexual couples in Zambia and Rwanda followed prospectively for HIV-1 transmission [9]. Remarkably, only 2 of 20 [10%; 95% confidence interval (CI) 1–32%] of the epidemiologically linked infections resulted from multivariant transmission, a finding attributed to the chronicity of infection in the virus positive partner, lower prevalence of comorbid conditions such as untreated tuberculosis or sexually transmitted infections, and the heterosexual route of transmission. Since the frequency of multivariant transmission in HSX in that study was substantially less than what we observed for MSM [2 of 20 HSX (10%, CI 1–32%) versus 10 of 28 MSM (36%, CI 19–56%); Fisher's exact p = 0.042, odds ratio 4.85, 95% CI 1.1 - inf], we performed a combined analysis of data from all four studies, which included 225 patients infected by HIV-1 subtypes A, B or C (**Table 3**). Again we found that the proportion of MSM subjects infected by more than one virus was substantially higher than for HSX [19 of 50 (38%) versus 34 of 175 (19%); Fisher's exact p = 0.008, odds ratio 2.5, 95% CI 1.2–5.3]. The MSM subjects were all infected with HIV-1 subtype B; a comparison to only the subset of HSX infections that were subtype B was still significant (Fisher's exact p = 0.01, odds ratio 2.9, 95% CI 1.2–7.1). The frequency of multiple infections in HSX was not statistically different among subtypes A, B and C nor was it different between males and females. Differences in the frequency of multivariant HIV-1 transmission in MSM versus HSX could not be accounted for by the numbers of sequences analyzed per subject nor by the clinical stage of subjects at the time of study. In the study by Haaland [9], the median number of sequences analyzed was 40, in Keele [10] it was 25, and in Abraham [8] it was 22. In the present study, the median number of sequences that we determined as part of our initial survey was 33 (**Table 2**). We used this lower number of sequences for statistical comparisons of single and multivariant transmissions in MSM versus HSX subjects in order to allow for comparability among the four studies. When, in this initial sequence set, we identified samples containing more than one transmitted/founder virus lineage, we went on to obtain additional sequences (as many as 89) in order to estimate more precisely the numbers of transmitted/founder viruses (**Table 2**). Increasing the numbers of sequences analyzed allowed for greater accuracy and precision in estimating the numbers of viruses transmitted in those individuals with many transmitted viruses (e.g., subjects 04013448, 04013171 and 701010068 in **Figs. 2D** **,** **6** and **7**), but it did not affect the discrimination between those subjects infected by one virus versus those infected by more than one virus. Finally, we found no significant correlation between the clinical stage of subjects at the time of plasma sampling and the numbers of transmitted/founder viruses identified in those samples: among the four studies, there was a total of 95 antibody negative subjects (Fiebig stages I–II) and 130 antibody positive subjects (Fiebig stages III–VI). Twenty subjects (21%) in the former group and 33 subjects (25%) in the latter group had evidence of productive infection by more than one virus, which was not significantly different (odds ratio 1.27; 95% CI 0.65–2.54; Fisher's exact p = 0.53).

**Table 3:** Multiplicity of HIV-1 infection in MSM vs heterosexual subjects.

Route of transmission	Study	Virus subtype	Total subjects	Single variant transmission		Multiple variant transmission				Number of variants		
			n	n	%	n	%	p value	odds ratio	median	range	Adjusted median^a^
Heterosexual	Keele [10]	B	79	65	82.3%	14	17.7%			1	1–4	2
	Abrahams [8]	C	69	54	78.3%	15	21.7%			1	1–5	3
	Haaland [9]	A and C	27	22	81.5%	5	18.5%			1	1–6	2
	Total		175	141	80.6%	34	19.4%			1	1–6	2
MSM	Keele [10]	B	22	13	59.1%	9	40.9%			1	1–6	3
	Li (*PLoS Path)*	B	28	18	64.3%	10	35.7%			1	1–10	3
	Total		50	31	62.0%	19	38.0%	0.008	2.5	1	1–10	3

a Adjusted median values are for multivariant transmissions only.

## Discussion

Previous studies used less precise methods for estimating multiplicity of HIV-1 infection in HSX and MSM subjects and reported widely varying results with a trend for higher multiplicities in MSM [15], [16], [17], [18], [19], [20], [21], [22], [24]. We report here new SGA-based determinations that show significant differences in the multiplicity of virus infection between the two risk groups: MSM were twice as likely as HSX subjects to become infected by more than one virus, with some MSM acquiring as many as 7 to 10 or more viruses. These findings are consistent with the higher epidemiological risk of HIV-1 acquisition in MSM compared with HSX and may be explained in part by the anatomical and immunohistological differences between the male and female genitourinary tracts and the lower intestine.

A limitation of the current study is that it represents a retrospective comparison of multivariant HIV-1 transmission among patient cohorts having different enrollment criteria and different behavioral risk assessments. It must be noted, however, that all study subjects from all cohorts were queried extensively with regard to potential HIV-1 infection risk behaviors. This included acutely infected subjects identified by cross-sectional screening methods [45], subjects enrolled prospectively into HIV-1 discordant couple [9] or Acute Infection Early Disease Research Program cohorts [46], and source plasma donors who became HIV-1 infected during a period of serial plasma collections [10]. The latter subjects, whom we studied anonymously, underwent exhaustive pre-enrollment interrogation for HIV and IDU risk behaviors according to a standardized FDA-approved protocol (http://www.fda.gov/BiologicsBloodVaccines/GuidanceComplianceRegulatoryInformation/Guidances/Blood/ucm073445.htm) that included a written questionnaire and interview inquiring about MSM and IDU activities, sex-for-money, sex with a partner who had sex-for-money, or sex with an individual known to be HIV positive. Source plasma donors also underwent serial laboratory testing for surrogate laboratory markers that could indicate injection drug use (e.g., liver transaminase elevations and hepatitis B or C nucleic acids or antibodies), and these markers were negative among qualified donors. Nonetheless, self-reporting of risk behaviors among paid plasma donors is imperfect [47], and it is possible that some subjects whom we categorized as belonging to the HSX risk group actually had additional risks such as IDU or MSM. However, even if this were the case, it would likely bias the findings in the HSX group toward a greater (not lesser) number of transmitted viruses [48]. Not surprisingly, when we excluded all source plasma donor subjects from our comparative analysis of multivariant HIV-1 transmission, the difference between MSM and HSX groups was still statistically significant [19 of 50 (38%) versus 25 of 119 (21%), respectively; Fisher's exact p = 0.03, odds ratio 2.3, 95% CI 1.04–5.02]. We conclude that within the limitations of self-reporting and surrogate marker testing, study subjects in the cohorts we examined were correctly assigned to HSX and MSM risk groups, differences in multiplicity of virus transmission between the two groups were significant, and overall study findings were unlikely to have been confounded by injection drug use. Future studies can benefit from a prospective trial design and a common behavioral and medical questionnaire [49], [50].

It is noteworthy that while multivariant HIV-1 transmission was twice as common in MSM than in HSX, still more than half of MSM subjects showed evidence of productive infection by just one virus. Moreover, the adjusted median (calculated from subjects with multivariant transmissions only) was 3 in MSM compared with 2 in HSX (**Table 3**). Even in the Fiebig II subject AD17, where we analyzed a total of 239 sequences (giving us a 95% probability of detecting a second transmitted/founder virus lineage at 1.25% prevalence), all of the sequences coalesced phylogenetically to a single virus, thus providing no evidence for transmission of more than one virus. Elsewhere, we have used 454 deep sequencing to analyze tens of thousands of sequences from three additional Fiebig stage II MSM subjects in whom SGA-direct sequencing suggested transmission and productive clinical infection by a single virus (Will Fischer, B.F.K., G.M.S. B.T.K., unpublished). Even with this greatly enhanced sensitivity of detection of minor sequences, we found no evidence of transmission by more than one virus in these subjects. Considered together, the findings of the present study, previously published studies [8], [9], [10], and work in progress (Will Fischer, B.F.K., G.M.S., B.T.K), all suggest that a substantial proportion of HSX and MSM patients acquire HIV-1 infection as a consequence of transmission and productive infection by literally one virion or one infected cell. The implication of this finding is that in order for a vaccine, microbicide or other prevention modality to be protective in this fraction of individuals, it need only prevent infection by a single virus or infected cell. Conversely, there is another subset of HSX and MSM subjects in whom the multiplicity of infection is higher. Since the proportion of such multiply infected individuals is far higher than would be expected from a Poisson distribution of independent, low frequency events (see Abrahams [8] for discussion), we suspect that biological events underlying virus transmission in these subjects compared with those infected by a single virus are different and that challenges faced by vaccines and microbicides in the higher multiplicity infection group may be higher.

Another interesting observation from the present study relates to viral recombination. Although recombination was not a primary study objective, the identification of two or more transmitted/founder genomes in acutely infected subjects gave us a unique opportunity to examine the dynamics and extent of recombination in primary HIV-1 infection. Five features of our study distinguish it from previous reports of HIV-1 recombination [39], [40], [41], [42]. First, we studied subjects at very early clinical stages following virus transmission (Fiebig stages II–V). Second, we used SGA-direct amplicon sequencing, which provides for a proportional representation of virus present in the plasma, including those that are recombinant [11]. Third, SGA eliminates *in vitro* recombination artifacts resulting from *Taq* polymerase-mediated template switching [11]. Fourth, SGA allowed us to identify the exact nucleotide sequences of full-length transmitted/founder virus *env* genes unambiguously and to distinguish these viruses and their progeny from viruses that contained even short regions of recombinant sequence. Fifth, SGA-direct sequencing of 3′ half genomes allowed us to examine recombination across the boundaries of *vif-vpr-tat-rev-vpu-env-nef*-LTR. **Figs 2D** **,** **6** and **7** illustrate examples of recombination and **Table 2** summarizes the findings in all multiply infected subjects. Seven of 9 subjects had evidence of recombination within gp160 *env* (one subject, AD77, could not be analyzed because of excessive virus diversity at a late Fiebig stage). The proportion of recombinants ranged from 0 of 30 sequences in subject 04013211 to 30 of 72 sequences (42%) in subject 701010068. In the latter subject, we amplified a longer fragment of the viral genome so as to include the 3′ half; this allowed us to compare recombination frequencies within gp41 (only), gp160 (only) or the full-length 3′ half genome. The proportion of recombinants in these three regions was 13/72 (18%), 30/72 (42%) and 63/72 (88%), respectively. Recombination breakpoints were more common in sequences flanking gp160 *env* than within *env* (**Fig. 7C**), a finding similar to that reported by Simon-Loriere and colleagues for HIV-1 inter-subtype recombination [42]. In subject 701010068, where 88% of sequences corresponding to only half the viral genome were recombinant, it is likely that nearly all of the full-genome sequences at this time point are recombinant. Since recombination requires an earlier infection event in which a cell is infected by two or more viruses, our findings suggest that in acutely infected humans at or near antibody seroconversion (Fiebig stages III/IV), a substantial fraction of productively infected cells are infected by more than one virus, a circumstance undoubtedly facilitated by initially high virus loads at a time when target cell availability is rapidly declining [51].

A final unique aspect to our study was its in-depth analysis of early virus replication kinetics (**Fig. 3**) and diversification (**Fig. 4**) in subject AD17 who was exposed to HIV-1 by receptive anal intercourse approximately 6 days before developing symptoms of the acute retroviral syndrome and 14–17 days before peak plasma viremia of 47,600,000 RNA molecules/ml. This exposure to HIV-1 was through a new sexual partner (AD18) whom we could prove by phylogenetic analysis was the source of subject AD17's acute HIV-1 infection (**Fig. 1**). Assuming a plasma viral load (vL) of 10 RNA copies/ml at the time of symptom onset 6 days after virus infection, then during the period between days 6 and 14, vL increased by a factor of ∼10^6^. This implies virus grew exponentially with growth rate r = 1.73/day, i.e. exp(1.73*8) ∼10^6^. This expansion rate is slower than the expansion rate calculated by Little [52] of 2.0/day but similar to that reported by Stafford [53] of 1.67/day. Subject AD17 began HAART on day 17, and between days 17 and 25, vL fell approximately 200-fold. Assuming HAART is nearly 100% effective [54], then the productively infected cell death rate, δ, can be calculated from the rate of vL decline as ln(200)/8 = 0.66/day. These values can then be used to estimate R_0_, the basic reproductive number, as (1+ r/δ) exp(rτ), where τ is the intracellular delay phase. If we ignore the delay phase, then R_0_ = (1+ r/δ) and the estimate of R_0_ is 3.6. However, if we include the delay phase and assume τ is one day, then R_0_ = 20.4. This is larger than the estimates in Stafford [53]. These data support the basic assumptions used in the development of our model of early HIV-1 evolution [10], [34], and the genomic integrity and replication competence of the full-length proviral clone pAD17.1 provide further corroboration of the model. Only four other transmitted/founder virus molecular clones have been described ([12] and J.S.G. and G.M.S., unpublished), and all of these correspond to HIV-1 subtype C viruses resulting from heterosexual transmissions. With the addition of the pAD17.1 clone, we now have molecular proviral clones representing male-to-male rectal transmission (pAD17.1), male-to-female vaginal transmission (pZM246F-10; pZM247Fv1; pZM247Fv2), and female-to-male penile transmission (pZM249M-1). All of these viruses are R5 tropic, replicate efficiently in activated human CD4+ T cells, but fail to replicate efficiently in monocyte-derived macrophages. Such molecular clones of transmitted/founder viruses should represent a rich resource for studying the biology of HIV-1 transmission and its prevention.

In summary, the findings presented here provide for the first time a comparative, quantitative view of the HIV-1 transmission event in two patient risk groups that dominate the HIV-1 pandemic. In doing so, they highlight both challenges and opportunities confronting candidate vaccines, microbicides, and other prevention modalities. Elucidation of the biological basis of single versus multivariant transmission in MSM and HSX could help advance prevention strategies [55], [56], [57], with quantitative analyses of transmitted/founder viruses representing a potentially valuable new endpoint in vaccine and microbicide trial design and assessment [5], [6], [49], [50].

## Materials and Methods

### Study Subjects

This study was conducted according to the principles expressed in the Declaration of Helsinki. It was approved by the Institutional Review Boards of the University of Alabama at Birmingham, Rockefeller University, Duke University, and the University of North Carolina. All patients provided written informed consent for the collection of samples and subsequent analysis. Blood samples were obtained from 28 subjects with acute HIV-1 infection and from chronically infected sexual partners of two of them. Blood specimens were generally collected in acid citrate dextrose and plasma separated and stored at −70°C. PBMCs were stored in vapor phase liquid nitrogen.

### Laboratory Staging

Plasma samples were tested for HIV-1 RNA, p24 antigen, and viral specific antibodies by a battery of commercial tests. These included quantitative Chiron bDNA 3.0 or Roche Amplicor vRNA assays; Coulter or Roche p24 Ag assays; Genetic Systems Anti-HIV-1/2 Plus O; and Genetic Systems HIV-1 Western Blot Kit. Based on these test results, subjects were staged according to the Fiebig classification system for acute and early HIV-1 infection [33].

### Viral RNA Extraction and cDNA Synthesis

For each sample, approximately 20,000 viral RNA copies were extracted using the Qiagen BioRobot EZ1 Workstation with EZ1 Virus Mini Kit v2.0 (Qiagen, Valencia, CA). RNA was eluted in 60 ul of elution buffer and subjected to first strand cDNA synthesis immediately by using the SuperScript III Reverse Transcriptase according to manufacturer's instructions (Invitrogen Life Technologies). Each first strand synthesis reaction included ∼10,000 or fewer vRNA molecules, 1X reverse transcription buffer, 0.5 mM of each dNTP, 5 mM DTT, 2 units/ul of RnaseOUT, 10 units/ul of SuperScript III reverse transcriptase and 0.25 uM of antisense primer. The cDNA syntheses were performed using antisense primers located at different genomic regions. The primers for synthesizing the cDNA of *env*, 5′ half genome (*U5, gag and pol)* and 3′ half genome (*vif*, *vpr, tat, rev, vpu, env, nef, U3 and R)* were env3out 5′-TTGCTACTTGTGATTGCTCCATGT-3′, 1.int.R1 5′-CTTGCCACACAATCATCACCTGCCAT-3′ and 1.R3.B3R 5′-ACTACTTGAAGCACTCAAGGCAAGCTTTATTG-3′, respectively. The reactions were incubated at 50°C for 60 min, followed by 55°C for an additional 60 min incubation. The reaction was heat-inactivated at 70°C for 15 min, and then treated with RNaseH at 37°C for 20 min. The synthesized cDNA was subjected to 1^st^ round PCR immediately or stored frozen at −80°C.

### Proviral DNA Extraction

Blood was collected from subject AD17 14–17 days following exposure to HIV-1 at Fiebig stage II. Genomic DNA was extracted from 1.3 million PBMCs using Qiagen Tissue DNA Extraction kit according to manufacturer's instructions.

### Single Genome Amplification

cDNA or genomic DNA was serially diluted and distributed in replicates of 8 PCR reactions in MicroAmp 96-well plates (Applied Biosystems, Foster City, CA) so as to identify a dilution where PCR positive wells constituted less than 30% of total number of the reactions. At this dilution, most wells contain amplicons derived from a single cDNA molecule. Additional PCR amplifications were performed using this dilution in 96-well reaction plates. PCR amplification was carried out in presence of 1x High Fidelity Platinum Taq PCR buffer, 2 mM MgSO4, 0.2 mM each deoxynucleoside triphosphate, 0.2 uM each primer, and 0.025 units/ul of Platinum Taq High Fidelity polymerase in a 20-ul reaction (Invitrogen, Carlsbad, CA). The nested primers for generating different genomic segments included: (1) full length *env*: 1^st^ round sense primer env5out 5′-TAGAGCCCTGGAAGCATCCAGGAAG-3′, 1^st^ round antisense primer env3out 5′- TTGCTACTTGTGATTGCTCCATGT-3′, 2^nd^ round sense primer env5in 5′-TTAGGCATCTCCTATGGCAGGAAGAAG-3′ and 2^nd^ round antisense primer env3in 5′-GTCTCGAGATACTGCTCCCACCC-3′; (2) 5′ half genome: 1^st^ round sense primer 1.U5.F1 5′- CCTTGAGTGCTTCAAGTAGTGTGTGCCCGTCTGT-3′, 1^st^ round antisense primer 1.int.R1 5′-CTTGCCACACAATCATCACCTGCCAT-3′, 2^nd^ round sense primer 2.U5.F2 5′-GTAGTGTGTGCCCGTCTGTTGTGTGACTC-3′ and 2^nd^ round antisense primer 2.int.R2 5′-CAATCATCACCTGCCATCTGTTTTCCATA-3′; (3) 3′ half genome: 1^st^ round sense primer 1.int.F1 5′- ACAGCAGTACAAATGGCAGTATT-3′, 1^st^ round antisense primer 1.R3.B3R 5′- ACTACTTGAAGCACTCAAGGCAAGCTTTATTG-3′, 2^nd^ round sense primer 2.int.F2 5′-TGGAAAGGTGAAGGGGCAGTAGTAATAC-3′ and 2^nd^ round antisense primer 2.R3.B6R 5′- TGAAGCACTCAAGGCAAGCTTTATTGAGGC-3′. PCR parameters were as follows: 94°C for 2 min, followed by 35 cycles of 94°C for 15 s, 58°C for 30 s, and 68°C for 4 min (*env*) or 5 min (5′ or 3′ half genomes), followed by a final extension of 68°C for 10 min. The product of the first-round PCR was subsequently used as a template in the second-round PCR under same conditions but with a total of 45 cycles. The amplicons were inspected on precast 1% agarose E-gel 96 (Invitrogen Life Technologies, Carlsbad, CA). All PCR procedures were carried out under PCR clean room conditions using procedural safeguards against sample contamination, including pre-aliquoting of all reagents, use of dedicated equipment, and physical separation of sample processing from pre- and post-PCR amplification steps.

### DNA Sequencing

Amplicons were directly sequenced by cycle-sequencing using BigDye Terminator chemistry and protocols recommended by the manufacturer (Applied Biosystems, Foster City, CA). Sequencing reaction products were analyzed with an ABI 3730xl genetic analyzer (Applied Biosystems; Foster City, CA). Both DNA strands were sequenced using partially overlapping fragments. Individual sequence fragments for each amplicon were assembled and edited using the Sequencher program 4.8 (Gene Codes; Ann Arbor, MI). All chromatograms were inspected for sites of mixed bases (double peaks), which would be evidence of priming from more than one template or the introduction of PCR error in early cycles. Any sequence with evidence of double peaks was excluded from further analysis.

### Sequence Alignments

All the sequence alignments were initially made with ClustalW and then hand-checked using MacClade 4.08 to improve the alignments according to the codon translation. Consensus sequences were generated for each individual. The full sequence alignment is available as a supplemental data file (www.hiv.lanl.gov/content/sequence/HIV/USER_ALIGNMENTS/Li) and sequences are deposited in GenBank (accession numbers: GU330247–GU331770).

### Sequence Diversity Analysis

Complete *env* sequences (n = 1307) were derived from 30 individuals, and 5′ (*U5, gag and pol*) and 3′ (*vif*, *vpu, tat, rev, env, nef, U3, and R*) half genome sequences (n = 188) were derived from PBMC and plasma at two different time points from subject AD17. We analyzed sequences for maximum sequence diversity and then visually inspected each set of sequences using neighbor-joining (NJ) and *Highlighter* tools (www.hiv.lanl.gov). Phylogenetic trees were generated by the neighbor-joining method using ClustalW or PAUP.

### Hypermutated Samples

Enrichment for APOBEC3G/F mutations violates the assumption of constant mutation rate across positions as the editing performed by these enzymes are base and context sensitive. Enrichment for mutations with APOBEC3G/F signatures was assessed using Hypermut 2.0 (www.hiv.lanl.gov). Sequences that yielded a p-value of 0.05 or lower were considered significantly hypermutated and excluded from subsequent analyses.

### Proviral DNA Cloning

To obtain an infectious molecular clone of the transmitted/founder virus of subject AD17, we amplified overlapping 5′ and 3′ half genomes from proviral DNA of earliest sample (day 14; 6/11/99) by single round PCR using Phusion Hot Start High-Fidelity DNA polymerase (Biolabs). Both fragments contained a complete LTR element and an overlap of 170 base pairs encompassing a unique *Sal*I restriction site. For the 5′ half genome, *U3-R-U5, gag, pol, vif*, *vpr* and *tat1* was amplified. For the 3′ half genome, *tat1, rev1, vpu, env, nef*, *tat2, rev2 and U3-R-U5* was amplified. The primers were designed to complement exactly the confirmed transmitted/founder sequence as determined by SGA-direct amplicon sequencing. The recognition sequences of *Mlu*I and *Not*I restriction enzymes were appended to the 5′ ends of the sense and antisense primers, respectively. Single round bulk PCR amplifications were carried out in the presence of 1X Phusion Hot Start HiFi buffer, 0.2 mM of each deoxynucleoside triphosphate, 0.5 uM of each primer, 3% final concentration of DMSO, and 0.02 units/ul of Phusion Hot Start High Fidelity polymerase in 50 ul reactions. The PCR product of each half genome was subjected to *Mlu*I and *Not*I digestion and gel purification and then independently cloned into the *Mlu*I-*Not*I site of TOPO XL vector (Invitrogen). The ligation mixture was transformed into XL2 Blue MRF competent cells and plated onto LB agar plates supplemented with 50 ug/ml of kanamycin and grown overnight at 30°C. Single colonies were selected and grown overnight in LB medium with same concentration of kanamycin at 30°C with constant shaking. Plasmid DNA was isolated and sequenced to confirm the identity of transmitted/founder sequences. The 5′ genome half was excised and cloned into 3′ TOPO XL vector by utilizing the *Mlu*I and *Sal*I restriction sites thereby generating the full length clone of the transmitted/founder provirus.

### Phenotypic Analyses

Replication competency of the full length molecular proviral clone AD17.1 was assessed using 293T cells, JC53BL-13 cells (NIH AIDS Research and Reference Reagent Program catalogue #8129, TZM-bl), activated primary human CD4+ lymphocytes, and monocyte-derived macrophages. Infectious virus stock generation, Env-pseudotyped virus stocks, titrations, cell infections and virus neutralization assays were performed according to methods previously described [12]. Virus controls (replication competent or Env-pseudotyped) included the HIV-1 macrophage-tropic strains YU2 and BaL, the non-macrophage tropic T-cell line-adapted strain NL4.3, the dual R5/X4 tropic strain WEAU1.60, and the xenotropic MuLV *env* [12]. The coreceptor inhibitors TAK779 and AMD3100 were obtained from the NIH AIDS Research and Reference Reagent Program (4983 and 8128). R5 and X4 tropism was assessed in both JC53BL-13 cells and in GHOST(3) cells that stably express CD4 along with CCR5 or CXCR4 or both or neither coreceptor.

### Recombination Analyses

Recombination was evaluated using GARD [58] and Recco [37] and by visual inspection of *Highlighter* plots. The minimum number of recombination events required to explain sequence datasets was estimated using the four-gamete method of Hudson and Kaplan [38] as implemented in DNASP v5.00.07 [59]. Recombinant sequences reported in Table 2 were identified by *Highlighter* analysis and confirmed by Hudson-Kaplan, GARD and Recco analyses.

### Mathematical Model

The model employed in the present study has been described [10], [12], [34] as have measured parameters of early virus expansion [52], [53], [54]. Under this model, with no selection pressure and fast expansion, one can expect small samples from homogeneous virus populations to have evolved from a founder strain in a star-like phylogeny with all sequences coalescing at the founder [10], [13]. Occasional deviations from a star phylogeny are, however, expected. The sampling of 10 sequences, for example, from a later generation of an exponentially growing population with six-fold growth per generation (R_0_ = 6) has about 3% chance of including at least one pair sharing the first four generations, a 19% chance of including sequences that share three, and a 75% chance of sharing two. Using a point mutation rate of about 1 per 5 generations for the full-length 9 kb HIV-1 genome [10], [34], there is about 75% chance of finding among ten sequences two that share one mutation, about 20% chance of finding two sequences that share a pair of mutations, and <2% chance of sharing more than that. These probabilities are slightly enhanced by early stochastic events that can lead to the virus producing less than six descendants in some generations but are diminished by the chances that mutations cause a fitness disadvantage that results in early purifying selection, as previously observed [10], [60]. We found examples of such early stochastic mutations leading to deviations from star phylogeny in several subjects (**Table 2**).

### Statistical Analyses and Power Calculations

We calculated the statistical significance of differences in rates of single versus multivariant HIV-1 transmission using Fisher's exact test. Differences were considered statistically significant at a value of p≤0.05. To estimate the likelihood of missing infrequently represented transmitted variants, we described previously a power study that estimated the probability of sampling low frequency plasma viral sequences [10]. In a sample set of at least n = 20, there is a 95% probability that a given missed variant comprises less than 14% of the virus population. For a sample set of 30, there is a 95% probability not to miss a variant that comprises at least 10% of the total viral population. And for a sample set of 80, there is a 95% probability not to miss a variant that comprises at least 4% of the total viral population.

We also considered the possibility that the number of transmitted/founder viruses detected could be influenced by the clinical stage (Fiebig stage) of the subjects at the time of virus sampling, because differences in virus replication rates could lead to increasing differences in virus frequencies with time. If this were the case and some viruses were outcompeted, the prediction would be that at later Fiebig stages the numbers of transmitted/founder virus lineages would be less than at earlier Fiebig stages. Our model (which is based on previously estimated parameters of an HIV-1 generation time of 2 days, a reproductive ratio [R_0_] of 6, and a reverse transcriptase error rate of 2.16×10^−5^ and assumes that the initial virus replicates exponentially infecting R_0_ new cells at each generation and diversifies under a model of evolution that assumes no selection) predicts that descendants of a transmitted virus at 45% replicative disadvantage compared to another transmitted virus, still have more than a 5% chance of occurring in a sample size of 20, ten generations (∼20 days) later. In humans, the eclipse period, defined as the time between HIV-1 transmission and first detection of virus in the plasma, has been estimated to be approximately 10–14 days, and the eclipse period plus Fiebig stages I and II, approximately 22–26 days [10], [33]. In the present study, the numbers of subjects in Fiebig stages I/II, III, IV and V were 14, 2, 6 and 6, respectively, and this relative distribution was similar in the three other studies included in the combined analysis [8], [9], [10]. As described in the main text, there was no significant correlation between clinical stage and multiplicity of infection (Fisher's exact p = 0.53).
